# Proteomic analysis of crop plants under abiotic stress conditions: where to focus our research?

**DOI:** 10.3389/fpls.2015.00418

**Published:** 2015-06-05

**Authors:** Fangping Gong, Xiuli Hu, Wei Wang

**Affiliations:** State Key Laboratory of Wheat and Maize Crop Science, Collaborative Innovation Center of Henan Grain Crops, College of Life Science, Henan Agricultural UniversityZhengzhou, China

**Keywords:** crop stress proteomics, abiotic stress, subcellular proteome, stress proteins, initial proteome stress response, post-translational modifications

## Introduction

Approximately 80% of human food is composed of crops, which are dominated by cereals that collectively make up 50% of global food production (Langridge and Fleury, 2011). Among cereal crops, rice, wheat, and maize provide approximately half of the calories consumed worldwide. Nevertheless, crop production is seriously hampered by influential abiotic stresses like drought, climate fluctuations, and salinity. It is estimated that up to 50–70% decline in crop productivity is attributed to abiotic stress (Mittler, 2006). Therefore, to ensure the security of global food production, it is essential to produce sustainable crop varieties that can adapt to climate variability, and to develop a broad spectrum of abiotic stress tolerant crops (Tester and Langridge, 2010). This has driven much research into the study of crop responses to abiotic stresses.

Proteomics has been successfully used to study abiotic stress responses in a wide range of crops (Abreu et al., 2013; Barkla et al., 2013; Ngara and Ndimba, 2014), especially rice (Kim et al., 2014), wheat (Komatsu et al., 2014), and maize (Benesova et al., 2012; Gong et al., 2014). It is generally envisioned that at this stage, proteomic-based discoveries in rice are likely to be translated into improving other crop plants against ever-changing environmental factors (Kim et al., 2014).

**Figure 1:**
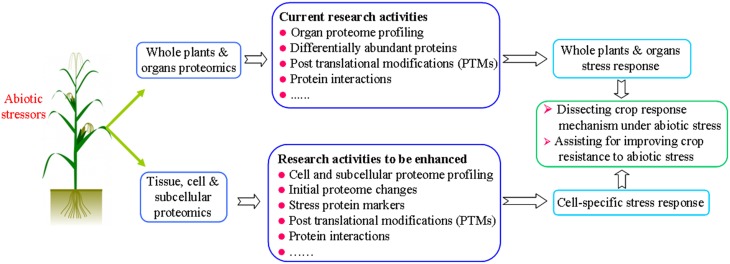
**A graphic summary on current research and future research in proteomic analysis of crop plants under abiotic stress conditions**.

Despite the potential role of proteomics to advance the study of stress tolerance in crops, thus far little useful information has been made available for crop improvement and breeding, even with the numerous proteomics studies undertaken in recent years. In our opinion, crop stress proteomics should be better focused on the following aspects: dissecting cell specific stress response (especially initial stress responses), identification of stress proteins, and the analysis of post translational modifications (PTMs) of proteins (Figure 1).

## Dissecting cell or tissue specific stress response

Understanding how plant cells sense and respond to abiotic stress is not only fundamental to our understanding of stress tolerance, but has the potential to yield novel approaches to improve crop productivity. Cellular proteomics plays an essential role in determining the functions of cellular compartments and the mechanisms underlying protein/gene targeting and trafficking.

Currently, numerous organ-specific proteomic analyses of abiotic stress in crops have contributed to our understanding of the response mechanisms of crops to abiotic stresses (Komatsu and Hossain, 2013). Obviously, the specifics of proteomic response to abiotic stress vary from tissue to tissue within a plant. Therefore, the crop stress response should be analyzed at a cellular or subcellular level, integrated with studies on whole plants, organs or tissues, to discriminate the specific responses of different cell types to abiotic stress. At present, cell or subcellular proteomic studies focus on relatively abundant, or easily isolated homogenous compartments (e.g., plastids, mitochondria, peroxisomes, and nuclei) mainly in Arabidopsis (Tanz et al., 2013), but also in rice, wheat, barley, maize (Huang et al., 2013; Millar and Taylor, 2014; Hu et al., 2015).

To increase the probability of identifying stress proteins (genes) from specific cells or tissues, an appropriate sampling method needs to be first developed to obtain relatively pure subcellular fractions from this material. A promising sampling method is laser capture microdissection (LCM), which can isolate specific cell types of interest from sectioned specimens of heterogeneous tissues under direct microscopic visualization with the assistance of a laser beam (Longuespée et al., 2014). LCM has been successfully used in transcriptome and microarray studies in maize (Nakazono et al., 2003; Rajhi et al., 2011) and rice (Suwabe et al., 2008; Kubo et al., 2013). Hopefully, combined with more sensitive protein staining technologies and more advanced mass spectrometers, LCM has the potential to promote crop stress proteomics at a cellular level. Another promising technique is free flow electrophoresis (FFE), which can isolate much purer membrane fractions and/or organelles. The FFE technique has been successfully applied in plant proteomics to the isolate tonoplast, mitochondria, plasma membranes, and Golgi apparatus (Barkla et al., 2013).

New approaches are beginning to enable the proteome to be analyzed at cell specific resolution. Not only are these studies leading to a better definition of the specialized proteomic behavior of certain cell types, but they also illustrate that information about proteome levels and responses gained from whole plants or organs can be misleading. This will surely help us to better understand the processes of crop stress acclimation and stress tolerance acquisition.

In addition, the studies on initial stress response of crops should be enhanced. Plant stress response consists of multiple phases, including an initial shock phase, an acclimation phase, a maintenance phase, an exhaustion phase, and/or a recovery phase (Kosová et al., 2011). Each phase of stress response is characterized by its unique transcriptome, proteome and metabolome changes. At 15 and 30 min after the onset of stresses (e.g., UV-B light, drought, and cold), the initial transcriptional changes in Arabidopsis are significant (Kilian et al., 2007). Currently, crop proteomic changes are often analyzed after several hours, even days after a stress onset (e.g., Meng et al., 2014). Thus, the initial proteome changes of crops under abiotic conditions should be further analyzed. These types of studies will give important insights in the signaling cascade activated immediately in crops in response to abiotic stresses. Sensitive proteomic approaches are capable of identifying low-abundance proteins (especially transcription factors and regulatory proteins) involved in the initial stress response in crops.

## Identification of stress proteins

The sequencing of major crops, especially rice, maize, and wheat represented a major breakthrough in crop proteomic research. For example, stress proteomic studies in maize have increased exponentially since 2009, after the release of the maize genome sequence (Schnable et al., 2009). Because knowledge of the genomic sequence alone does not indicate how a plant interacts with the environment, and not all open reading frames correspond to a functional gene (Ribeiro et al., 2013), proteomic approaches are critical to understand plant mechanisms of stress tolerance. A main aim of stress proteomics in crops is to identify stress proteins which can potentially be used for crop improvement and breeding.

The stress-tolerant phenotype in crops is a result of differential expression of unique proteins in resistant cultivars to protect them during stress periods. To develop better crop plants for sustainable food production, proteomic discovery of these unique stress proteins to further understand the stress-tolerance mechanisms at the molecular level is very important. We can potentially modify these key stress proteins to enhance a crops abiotic stress tolerance. A potential role for crop stress proteomic studies could be the identification and further characterization of key proteins underlying crop tolerance to a given abiotic stress, which can then be used as protein biomarker of a given stress. In such abiotic stress studies, it is common to analyze proteomes by contrasting stressed crop plants against control ones, attempting to correlate changes in protein accumulation with the phenotypic response (Abreu et al., 2013).

Rapid progress in proteome profiling methodologies, such as iTRAQ, DIGE, and high-resolution tandem mass spectrometry has enabled a more accurate comparison of crop stress responses and can detect more differentially abundant proteins than prior analyses. Unfortunately, ascribing a probable function to a newly identified stress responsive protein can be difficult in crops. Unlike Arabidopsis, it is difficult to determine the number of experimentally characterized genes in public databases for many crops. This is mainly due to the lack of high quality functional annotations for many crop genomes. For example, the maize genome sequence contains approximately 40,000 genes (Schnable et al., 2009), but little is known about the function of most genes. A search for maize protein sequences using the keyword “maize” retrieved 262,228 entries at NCBI and 85,389 entries in UniProt (14 May, 2015). This is indicative of the high level of redundancy and repetition, particularly in the NCBI database. In the UniProt database, only 840 maize protein entries have been reviewed (14 May, 2015), with most entries listed as “uncharacterized protein.” Likewise, in rice, only 1% of the protein-coding genes have had a functional annotation based on experimental evidence (Rhee and Mutwil, 2014). Given this situation, the experimental validation of stress proteins and their role in stress tolerance is very important to bridge the gap between proteomic discovery of stress proteins and the selection of potential target proteins for future crop improvements.

More attention should be paid to up-regulated proteins in crop stress proteomics. In plants, translation efficiency can change dramatically in response to abiotic stress, leading to a massive bias in the pool of mRNAs that are actively translated (Mustroph et al., 2009; Juntawong et al., 2014). This might be related to the importance of stress- associated proteins that are required to recalibrate cellular metabolism. Thus, up-regulated proteins are more important for crops to adapt to a stressful environment compared to down-regulated proteins; an important point when considering crop stress tolerance breeding. Of course, down-regulated proteins are also likely to contribute to an acquisition of enhanced plant stress tolerance. For example, some secondary metabolism related proteins affected by stress would likely decrease to conserve energy (Ghosh and Xu, 2014). In addition, due to discordant protein and mRNA expression, especially in plants (Vélez-Bermúdez and Schmidt, 2014), it is essential to identify up-regulated stress proteins rather than mRNA in order to better identify candidates which could be used for crop improvement.

Finally, it is worth noting that much of the stress proteomic research has been performed in laboratories under controlled conditions and relied on screening whole crops for their ability to survive severe stress. The effects of plant growth and gene expression in response to stress can be highly dose-responsive, indicating the existence of sensitive machinery in the plant for assessing the stress level and fine-tuning molecular responses (Claeys et al., 2014). Therefore, many proteomic analysis of abiotic stress in crops can be misleading and may not be useful.

## Analysis of PTMs of proteins

PTMs can affect protein function, interactions with other proteins, subcellular targeting, and stability. In crop stress proteomics, the identification and quantification of PTMs will contribute the detailed functional characterization of a protein and will likely assist in our understanding of crop stress acclimation and stress tolerance acquisition.

Large-scale proteomics studies have revealed that PTMs are more widespread than previously estimated. For example, up to two-thirds of the metabolic proteins in yeast may be affected by protein phosphorylation (Breitkreutz et al., 2010). In Arabidopsis, PTMs include phosphorylation (Heazlewood et al., 2008; Vialaret et al., 2014), *N*-linked glycosylation (Zielinska et al., 2012), ubiquitination (Kim et al., 2013), methionine oxidation (Marondedze et al., 2013), *S*-nitrosylation (Fares et al., 2011), and acetylation (Finkemeier et al., 2011).

Few proteomic studies have been performed to specifically characterize PTMs in crops under abiotic stress; this includes the analysis of phosphorylation during salt and water stresses in maize (Zörb et al., 2010; Bonhomme et al., 2012; Hu et al., 2013) and characterization of protein glycosylation in soybean roots under flooding (Mustafa and Komatsu, 2014).

It is expected that in certain cases PTMs play a more important role than protein abundance changes. Thus, the analysis of PTMs of stress-responsive proteins in crops should be strengthened; however the ability to routinely identify and quantify PTMs represents a grand challenge in the field of proteomics (Heazlewood, 2011). In conjunction with improvements in methodological approaches, it would be expected that the study of PTMs will become more common in the area of crop stress proteomics in the future.

## Concluding remarks and outlook

Proteomics has an important role to play in assisting our understating at the molecular level of how crops respond to abiotic stress. In-depth proteomic analysis of crop stress responses will be essential for future crop improvements. Though proteomic characterization of cell and tissue specific stress responses, stress proteins and PTMs is still a difficult undertaking in crops, the development of more sensitive methodologies, particularly for the cell specific analysis of the proteome will be crucial for understanding stress responses at the cellular level. In addition, rapid advances in high-throughput omics technologies (e.g., proteomics, transcriptomics, genomics, and metabolomics) will make it possible to use a systems biology approach to understand crop responses to abiotic stresses.

### Conflict of interest statement

The authors declare that the research was conducted in the absence of any commercial or financial relationships that could be construed as a potential conflict of interest.
